# Correlation of Co-Morbidities with Symptom Severity of Children with Autism Spectrum Disorder: A Cross-Sectional Survey

**DOI:** 10.3390/nu16172960

**Published:** 2024-09-03

**Authors:** Mingyang Zou, Yilin Zhang, Dexin Li, Shengqi Li, Jingyi Hu, Ya Gao, Zeyu Cheng, Shidan Liu, Lijie Wu, Caihong Sun

**Affiliations:** 1 Department of Children’s and Adolescent Health, College of Public Health, Harbin Medical University, Harbin 150081, China; mingyangshine@hrbmu.edu.cn (M.Z.); 2022020182@hrbmu.edu.cn (Y.Z.); ldx19940611@163.com (D.L.); 2023020096@hrbmu.edu.cn (J.H.); 2023020093@hrbmu.edu.cn (Y.G.); 2023020090@hrbmu.edu.cn (Z.C.); 2023020092@hrbmu.edu.cn (S.L.); hydwlj@ems.hrbmu.edu.cn (L.W.); 2 Beijing Normal University—Hong Kong Baptist University United International College, Zhuhai 519087, China; s230016023@mail.uic.edu.cn; 3 Department of Developmental Behavioral Pediatrics, The Sixth Affiliated Hospital of Harbin Medical University, Harbin 150023, China

**Keywords:** autism spectrum disorder, comorbidities, serum nutrient levels, diet partiality, sleep disturbances

## Abstract

This study aims to identify potential correlations of the severity of symptoms of children with autism spectrum disorder (ASD) with serum nutritional levels, body composition indicators, diet partiality, and sleep disturbances. The cohort of this cross-sectional study included 120 children with ASD and 110 typically developing (TD) children to assess symptoms of ASD, and to measure serum levels of vitamins and minerals and the body composition values. Diet partiality and sleep disturbances were assessed by administering questionnaires. The serum levels of folic acid, copper, and vitamin B were lower in children with ASD than in TD children, while magnesium and homocysteine were higher (*p* < 0.05). Children with ASD had greater chest circumference, abdominal skinfold thickness, and body mass index (BMI) than TD children (*p* < 0.05), and higher prevalences of diet partiality and sleep disturbances (*p* < 0.001). Lower vitamin A levels and higher vitamin D levels were related to social impairment in children with ASD. Moreover, there were significantly positive correlations of BMI, chest circumference, diet partiality, and sleep disturbances with severity of ASD symptoms (*p* < 0.05). Collectively, rational nutritional supplementation, dietary management, and behavioral interventions are essential for children with ASD.

## 1. Introduction

Autism spectrum disorder (ASD) is a complex neurodevelopmental condition characterized by limited social communication and repetitive and restricted interests and behaviors [1,2]. According to the latest data released by the American Center for Disease Control and Prevention, the overall prevalence of ASD in 8-year-old children is 1/36 [3]. As one of the most common emerging pediatric diseases, ASD has attracted worldwide attention as a spectrum that can range from very mild to severe [4]. Recent studies have mostly focused on the core symptoms of ASD, while relatively few have investigated potential correlations of comorbidities of children with ASD with core symptoms, nutritional imbalances, mealtime issues, and sleep disturbances.

Due to typical repetitive behavioral traits, children with ASD have notable dietary restrictions, preferring a limited variety of food and being reticent to food in new environments and forms, which may be linked to vitamin and mineral deficiencies [5]. Preschool children with ASD are at particular risk for the highly inadequate intake of vitamins D and E, calcium, and magnesium, as well as the moderately inadequate intake of vitamin C, folate, and iron [6]. Inadequate nutrient intake inevitably leads to nutrient imbalances in the body. Serum levels of vitamins and minerals, such as calcium, copper, and vitamins D, B6, and B12, are reportedly lower in children with ASD [7,8], which are accompanied by the accumulation of homocysteine [9]. Nutrient imbalances also influence physiological functions and, ultimately, the symptoms of ASD [7,10]. In addition, children with ASD may be at higher risks of overweight and obesity than typically developing (TD) children [11], which may be linked to poor eating habits and nutrient imbalances [12]. Furthermore, sleep disturbances can disrupt biological rhythms and impair brain function in children [13]. ASD has also been associated with sleep disturbances due to internalizing and externalizing behaviors and somatic disorders in children with ASD, which could influence sleep [14]. Many studies have found that sleep disturbances impact the symptoms of ASD, such as limited social skills and communication difficulties [15].

Since nutrient levels, body composition, diet, and sleep problems are potentially associated with the symptoms of ASD, the aim of the present study was to assess the impacts of serum nutrient levels, physical dimensions, diet partiality, and sleep disturbances on the core symptoms of ASD in preschool children to provide a scientific basis for clinical monitoring and intervention.

## 2. Materials and Methods

### 2.1. Participants

The study cohort included 120 children with ASD (aged 2–7 years) residing in Harbin, China. ASD was diagnosed by two independent specialist clinicians based on the diagnostic criteria of the *Diagnostic and Statistical Manual of Mental Disorders, Fifth Edition* [16] in addition to the Autism Diagnostic Observation Schedule and the Autism Diagnostic Interview—Revised [17]. The exclusion criteria were (a) a history of other developmental or psychiatric disorders, genetic disorders with comorbid autism, Rett syndrome, cerebral palsy, chronic seizures, and other congenital diseases, (b) recent infection, and (c) use of high-dose vitamin or mineral supplementation.

The control group included 110 TD children (aged 2–7 years) residing in Harbin, China, with no signs of neuropathology or any pathology known to impact vitamin or mineral homeostasis, and not receiving high-dose vitamin or mineral supplementation.

Data from the ASD and TD groups were collected in winter in 2019 and 2020. The research protocol was approved by the Research Ethics Committee of the Children’s Hospital of Fudan University ([2012] No. 185) and written informed consent was obtained from the parents of eligible children prior to enrollment in the study. All participants recruited for the purpose of the research received oral feedback and a written summary of the results of evaluation without any payment.

### 2.2. Measurements

Demographic characteristics (age, sex, ethnicity, resident type, maternal abnormalities during pregnancy, maternal education level, paternal education level, family structure, sleeping habits, and eating habits) were collected from the guardian of each participant (Appendix A). The sleep habits questionnaire consisted of six aspects of sleep disorders: night awakening, daytime sleepiness, parasomnias, sleep anxiety, prolonged time to fall asleep, and poor sleep habits. Children who exhibited any of these behaviors were considered to have this kind of sleeping disorder, while those exhibiting 4–6 of these behaviors were considered to have sleep disturbances. Diet partiality was assessed by asking the questions “Does your child have a significant diet partiality?” and “Please list the types of food your child resists eating”.

The Autism Behavior Checklist (ABC), Childhood Autism Rating Scale (CARS), and Social Responsiveness Scale (SRS) were used to evaluate symptoms of ASD. Higher scores of the ABC, CARS, or SRS indicate more autistic symptoms.

(a) The ABC is used for behavioral examination, auxiliary diagnosis, and screening of ASD. A cut-off total score greater than 67 points indicates a high probability of ASD, while scores of 53–67 indicate potential ASD [18].
(b) The CARS is used to evaluate the symptoms and duration of ASD. Scores of 30–36 points indicate moderate ASD and scores > 36 points indicate severe ASD [19].
(c) The SRS is used to screen the social behavior of children and adolescents with ASD to determine the severity of social disorders [20].

### 2.3. Lab Measurements

Fasting blood samples (5 mL) were collected by venipuncture at 08:00 h at room temperature (~21 °C) and centrifuged at 3000 rpm for 10 min to separate the serum and blood cells. Serum levels of serotonin and vitamins A and E were measured by high-performance liquid chromatography (DGU-20As; Shimadzu Corporation, Kyoto, Japan). Serum vitamin D levels were measured using an immunoassay (Architect 25-OH vitamin D assay with the Architect i2000SR immunoassay analyzer; Abbott Diagnostics, Lake Forest, IL, USA). Serum levels of homocysteine, folic acid, and vitamin B12 were measured with chemiluminescence microparticle immunoassay kits (Abbott Ireland Diagnostics Division, Longford, Ireland). Serum levels of calcium, magnesium, iron, copper, and zinc were determined by atomic absorption spectrophotometry.

### 2.4. Physical Examination

To avoid the influence of different time periods and seasons on physical development and the results of biochemical tests, the physical examinations were scheduled at the same time as the biochemical tests. All participants were evaluated in the early morning on an empty stomach using a body composition tester (InBody J30; Imu Medical Devices Ltd., Shanghai, China). The body composition indicators included height, weight, fat mass, fat free mass, total body water content, protein levels, inorganic salt levels, and skeletal muscle mass. In addition, head, chest, waist, and hip circumferences were measured by professional staff members using the multifunctional ruler of the Child Physical Development Test.

### 2.5. Statistical Analysis

Statistical analysis was performed using IBM SPSS Statistics for Windows (version 26.0; IBM Corporation, Armonk, NY, USA). The data are presented as the mean ± standard deviation, median (interquartile range), or frequencies and percentages. The normality of the distribution of variables was assessed using the Kolmogorov–Smirnov goodness-of-fit test. Differences between the two groups were assessed with the Student’s *t*-test, Mann–Whitney U-test, or chi-square test, as appropriate. Partial correlation analysis was used to determine the relationship between serum nutrient levels and clinical traits with adjustments for demographic characteristics. A two-sided probability (*p*) value > 0.05 was considered statistically significant.

## 3. Results

### 3.1. Participant Characteristics

The ASD group included 95 boys and 25 girls with a mean age of 4.06 ± 0.98 years, while the TD group included 81 boys and 29 girls with a mean age of 4.34 ± 0.88 years. The general characteristics of the children enrolled in this study are shown in Table 1. There were significant differences in age (*p* = 0.024), resident type (*p* < 0.001), maternal education level (*p* < 0.001), and paternal education level (*p* < 0.001) between the two groups, but not in the sex ratio, ethnicity, abnormal condition during pregnancy, or family structure. The mean scores of the ABC and CARS of the ASD group were 51.36 ± 27.05 and 28.24 ± 4.75, respectively. The SRS score was higher for the ASD group than the TD group (86.38 ± 22.02 vs. 39.47 ± 15.46, respectively, *p* < 0.001).

### 3.2. Serum Nutrient Levels and Body Composition

As shown in Table 2, the serum levels of folic acid (*p* = 0.007), copper (*p* = 0.001) and vitamin B12 (*p* < 0.001) were significantly lower in the ASD group than the TD group, while the serum levels of magnesium (*p* < 0.001) and homocysteine (*p* = 0.047) were significantly higher. As shown in Table 3, chest circumference (*p* = 0.001), abdominal skinfold thickness (*p* = 0.002), and body mass index (BMI) (*p* < 0.001) were greater in the ASD group than the TD group.

### 3.3. Sleep Disturbances and Diet Partiality

**Figure 1:**
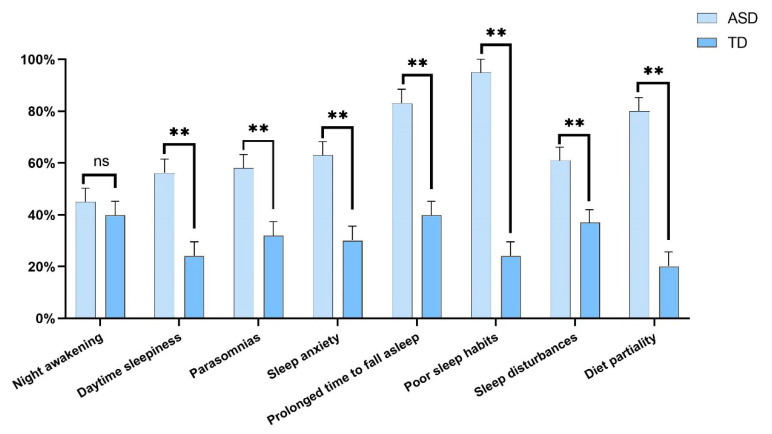
Comparisons of sleep disorders and diet partiality between the ASD and TD groups. ns indicates *p* > 0.05; ** indicates *p* < 0.01.

As shown in Figure 1, there were notable differences in sleep disorders and diet partiality between the ASD and TD groups. Children with ASD exhibited more daytime sleepiness (*p* < 0.001), parasomnias (*p* < 0.001), sleep anxiety (*p* < 0.001), prolonged time to fall sleep (*p* < 0.001), and poor sleep habits (*p* < 0.001) than TD children. There was no significant difference in night awakening between the two groups (*p* = 0.256). Children with ASD had more severe sleep disturbances (*p* < 0.001) and more severe diet partiality (*p* < 0.001).

### 3.4. Correlation Analyses in Children with ASD

**Figure 2:**
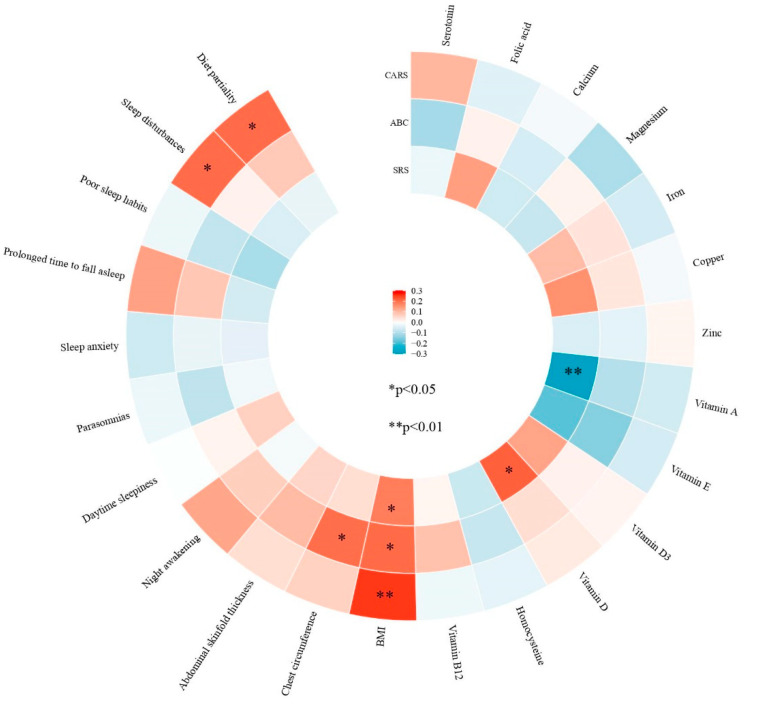
Associations of severity of ASD symptoms with serum nutrient levels, body composition, sleep disorders, and diet partiality. The *p*-values were adjusted for age, sex, resident type, maternal education, and paternal education.

The correlations of the clinical traits of ASD (indicated by ABC, CARS, and SRS scores) with serum nutrient levels, body composition, sleep disturbances, and diet partiality after adjusting by demographic characteristics are presented in Figure 2. Serum vitamin A levels were significantly negatively correlated with the total SRS score (*r* = 0.302; *p_adj_* = 0.001), while serum vitamin D levels were significantly positively correlated (*r* = 0.220; *p_adj_* = 0.018). However, there were no significant associations of the other serum nutrient levels with the ABC and CARS scores. There was a significant positive correlation between BMI and the SRS, ABC, and CARS scores (*r* = 0.185, 0.207, and 0.257; *p_adj_* = 0.044, 0.024, and 0.005, respectively), and a significant positive correlation between chest circumference and ABC scores (*r* = 0.203; *p_adj_* = 0.03). CARS scores were higher for autistic children with diet partiality (*r* = 0.206; *p_adj_* = 0.026) and sleep disturbances (*r* = 0.206; *p_adj_* = 0.026).

## 4. Discussion

The results of this cross-sectional study found that children with ASD have comorbid symptoms of diet partiality and sleep disturbances and are at risk of developing nutrient imbalances and excess weight gain, which are associated with the severity of ASD symptoms.

As essential nutrients, minerals play important roles in the regulation of metabolism and various biochemical reactions. Also, minerals are closely related to the pathogenesis of ASD, and children with ASD have multiple mineral imbalances [21,22]. Copper is a cofactor of several essential enzymes of the central nervous system, and the dysregulation of copper homeostasis has been linked to several neurodegenerative diseases [23]. In the present study, the serum levels of copper were relatively low in the ASD group, although there was no association between copper levels and ASD symptoms, in agreement with a previous report that children with ASD had low levels of copper in the hair [22]. Interestingly, a recent study of children residing in Jilin Province showed that serum copper levels were higher in the ASD group than the TD group and positively correlated with the severity of ASD symptoms [10]. Similarly, magnesium plays a crucial role in neurotransmission and neuromuscular conduction, and also protects against overexcitation that may lead to neuronal cell death (excitotoxicity) and a wide range of neurological disorders [24]. In agreement with the results of the present study, previous reports noted higher serum magnesium levels in children with ASD than TD children, suggesting a potential dietary relationship [25,26]. The high correlation between serum levels of metal elements and dietary intake suggests that low serum copper and magnesium levels can be used as indicators of inadequate intake or lack of proper intestinal absorption of nutrients in children with ASD due to limited diets.

Folic acid, vitamin B12, and homocysteine play key roles in the one-carbon metabolic pathway and methionine cycle, which regulate DNA synthesis, cell proliferation, and inflammation responses in the brain [27]. The results of the present study also confirmed that children with ASD had aberrant folate-related metabolism [28]. The results of this study are in line with previous reports of lower folic acid and vitamin B12 levels, as well as elevated homocysteine levels, in the ASD group as compared to the control group [7,29,30]. Among the vitamins related to neurodevelopment, the fat-soluble vitamins A and D are essential micronutrients that regulate brain development, neurogenesis, and neurons [31,32]. Deficient serum levels of vitamins A and D are risk factors for ASD, while supplementation can ameliorative impaired behaviors in children with ASD [33,34]. We did not find differences in the levels of vitamin D3 and D among children with ASD in the present study; some of the existing literature agreed with our findings, but some disagreed. Studies from California showed no association between maternal and neonatal 25-hydroxyvitamin D and ASD risk [35], while studies from Netherlands and China indicated that higher concentrations of 25-hydroxyvitamin D was linked to lower risk of ASD [36,37,38]. In the present study, lower vitamin A levels and higher vitamin D levels were related to social impairment in children with ASD. Notably, vitamin A plays a synergistic role in the bioactivity of vitamin D and is closely associated with the expression levels of retinoic acid X receptors, while vitamin D levels are influenced by geographic location and latitude as well as exposure to ultraviolet B light [39]. Since all participants in this study resided in the north-eastern region of China, where winter sunshine is shorter and dietary preferences, such as low consumption of fresh vegetables, are influenced by environmental factors, the serum levels of vitamins A and D were relatively deficient in both groups. The 2024 Chinese Expert Consensus on the Clinical Application of Vitamin A and Vitamin D in Children defines deficient serum levels of vitamins A and D (25-OH) as <0.70 gmol/L and <30 nmol/L, respectively. With regard to the contradictory results of positive correlation of vitamin D with SRS scores, it might be attributed to the interaction with vitamin A [40], the status and function of vitamin D receptors [41], or other forms of vitamin D metabolic abnormalities [42]. Although nutrient levels are generally abnormal in ASD children, there is no consensus among recent studies, which may be related to differences in ethnicity, geography, and the ages of the children. In addition, the complex developmental etiological mechanisms of ASD may be responsible for the lack of consensus. Furthermore, the tangled interaction between limited food selectivity [43], nutrient intake and metabolism [44], the core impairments of ASD, and nutritional imbalances remains to be more clearly elucidated. All in all, the latest studies suggested an association between nutrient levels and ASD.

The association between ASD and obesity should not be underestimated. A meta-analysis showed that 17% of children with ASD are obese, with a relative risk of obesity of 1.58, which was higher than for healthy controls [45]. When the fat component is taken into account, the obesity rate in children with ASD can be up to 47.5%, possibly due to a high degree of food rejection, limited food choice, and aggressive behavior at meal times [43]. Children with ASD have a higher risk of excess weight gain and eating problems, consistent with the findings of the present study that children with ASD had a greater chest circumference and BMI with severe diet partiality, which were related to more severe symptoms of ASD. Although preschoolers with ASD in the current study were not necessarily obese, obesity tends to peak during puberty. It remains unclear whether obesity is causally associated with ASD. Nonetheless, diet partiality and limited physical activities derived from core impaired behaviors are associated with an increased risk of obesity in children with ASD [43,46]. Moreover, ASD has been associated with a higher prevalence of constipation, likely due to reduced microbiota and abundances of related metabolites in the gastrointestinal tract [47], as well as aberrations in obesity-related genes [48]. Therefore, autistic children should be encouraged to regularly participate in physical activities and adopt healthy dietary habits to decrease the risk of obesity.

Sleep disturbances are common in children and even in newborns with ASD [49,50,51,52]. In the present study, sleep disturbances in ASD children were mainly reflected as daytime sleepiness, parasomnias, sleep anxiety, prolonged time to fall asleep, and poor sleep habits. Moreover, the prevalence of prolonged time to fall asleep and poor sleep habits was greater than 80%, which may be associated with disruptions to the sleep–wake cycle and decreased sleep-related brain neurotransmitters (e.g., melatonin, cortisol, and gamma-aminobutyric acid) [53]. Western studies reported the prevalence of sleep disorders ranging between 53% and 81% among preschool children with ASD using the Children’s Sleep Habits Questionnaire, and ASD children with poor sleep quality exhibited more aggressive, anxious, self-harming, and inattentive behaviors, along with more severe core symptoms of ASD [54,55]. Furthermore, the latest meta-analysis showed that sleep problems in children and adolescents with ASD were associated with core symptoms, especially sleep anxiety and sleep-onset delay, which were significantly related to restrictive and repetitive behaviors [56]. Although there were differences in terms of methodology, respondents, and methods of analysis, the previous studies and our study in Chinese children draw the consistent finding that there is a strong association between sleep disturbances and symptoms of ASD. However, since sleep disturbances are often not identified in clinical practice, the impact of these maladaptive behaviors on daytime rehabilitation should be considered. In addition, the retrospective studies suggested that sleep–wake rhythm abnormalities in neonates and the misalignment and/or shift of the circadian rhythm in infants (<3 years of age) were potential risk factors for the future development of ASD [51,57]. A prospective, longitudinal observation revealed that difficulties with sleep onset in the first year of life preceded ASD diagnosis [52]. Therefore, sleep problems are not only linked to symptoms, but may be deeply associated with ASD pathogenesis.

### Limitations and Future Research Directions

There were some limitations to this study that should be addressed. First, the subjects of this study were all children with ASD from designated rehabilitation institutions in Heilongjiang Province, which limits coverage and representativeness. Second, information on the diets and food choices of children with ASD was not collected. The dietary differences among participants may have influenced the results. Therefore, standardized diets should be considered for future studies. Finally, since this was a cross-sectional study with a limited sample size, causality could not be determined.

## 5. Conclusions

These findings suggest that children with ASD have nutrient imbalances, higher BMI, diet partiality, and sleep disturbances that are likely to lead to the exacerbation of ASD symptoms. Hence, comprehensive assessments of comorbid symptoms and individualized interventions are recommended for certain subgroups of children with ASD.

## Figures and Tables

**Table 1:** Demographic characteristics of the ASD and TD groups.

General Characteristics	ASD $\left(N=120\right)$	TD $\left(N=110\right)$	p
Age	4.06 ± 0.98	4.34 ± 0.88	**0.024**
Sex (male) [n (%)]	95 (79.2)	81 (73.6)	0.323
Ethnicity (Han) [n (%)]	111 (92.5)	101 (91.8)	0.847
Resident (Urban) [n (%)]	92 (66.7)	110 (100)	**<0.001**
Maternal abnormalities during pregnancy [n (%)]	55 (45.8)	38 (34.6)	0.081
Mother’s education [n (%)]			
Illiterate/elementary/middle school	37 (30.8)	3 (2.7)	**<0.001**
High school	14 (11.7)	6 (5.5)	
College or above	69 (57.5)	101 (91.8)	
Father’s education [n (%)]			
Illiterate/elementary/middle school	34 (28.3)	2 (1.8)	**<0.001**
High school	13 (10.8)	7 (6.4)	
College or above	73 (60.8)	101 (91.8)	
Family structure (Nuclear family) [n (%)]	58 (48.33)	53 (48.18)	0.982

Bold values indicate *p* < 0.05.

**Table 2:** Comparisons of serum nutrient levels between the ASD and TD groups.

Types	ASD $\left(N=120\right)$	TD $\left(N=110\right)$	t/Z	p
Serotonin	127.5 (81.75–172.75)	133 (92.5–167)	−0.18	0.858
Folic acid	11.33 ± 3.29	12.48 ± 3.10	−2.711	**0.007**
Calcium	92.9 (88.25–95.98)	92.8 (87.2–98.4)	−0.816	0.414
Magnesium	19.86 ± 1.81	18.92 ± 1.54	4.266	**<0.001**
Iron	1133 (873–1445.25)	1087 (844.5–1346.5)	−0.442	0.658
Copper	1054.5 (929.75–1170.5)	1123 (998.5–1301)	−3.303	**0.001**
Zinc	815.5 (728.5–887.75)	810 (759–876)	−0.465	0.642
Vitamin A	0.36 ± 0.08	0.35 ± 0.07	0.565	0.572
Vitamin E	8.9 (7.6–10)	8.4 (7.3–9.95)	−1.162	0.245
Vitamin D3	18.55 (12.9–27.75)	20.5 (15.25–28.05)	−0.785	0.433
Vitamin D	19.75 (13.4–28.05)	21.2 (15.9–28.2)	−0.769	0.442
Homocysteine	5.66 (4.953–6.523)	5.39 (4.63–6.125)	−1.984	**0.047**
Vitamin B12	736.52 (550.4–1039.4)	919 (710.4–1131.4)	−3.49	**<0.001**

Bold values indicate *p* < 0.05.

**Table 3:** Comparisons of body composition between the ASD and TD groups.

Body Dimension	ASD $\left(N=120\right)$	TD $\left(N=110\right)$	t/Z	p
Head circumference	50.5 (49.5–51.5)	50.5 (49.35–51.55)	−0.259	0.796
Chest circumference	53.5 (51.8–56.8)	52.6 (50.55–54.65)	−3.377	**0.001**
Waistline	49 (47.5–52.43)	49.5 (47.25–51.7)	−0.016	0.987
Hip circumference	55 (52.05–59)	55.7 (52.2–58.3)	−0.176	0.861
Height	104.2 ± 8.66	104.78 ± 7.8	−0.535	0.593
Weight	18.50 ± 4.11	17.69 ± 3.25	1.646	0.101
Triceps skinfold thickness	9 (8–11)	10 (8.5–11.5)	−0.756	0.45
Subscapular skinfold thickness	6 (5–7)	6 (5.5–7)	−0.174	0.862
Abdominal skinfold thickness	6 (5–8.5)	5.5 (4.5–7)	−3.028	**0.002**
Total Body Water	10.35 (8.75–11.79)	9.8 (8.75–11.2)	−1.557	0.12
Protein	2.7 (2.3–3.1)	2.6 (2.3–2.9)	−1.145	0.135
Minerals	0.87 (0.74–1.06)	0.86 (0.74–0.98)	−0.75	0.454
Body Fat Mass	3.95 (3.1–5.3)	4.1 (3.2–5.1)	−0.21	0.833
Soft Lean Mass	13.2 (11.25–15.18)	12.6 (11.2–14.3)	−1.576	0.115
Fat Free Mass	14.05 (11.83–16.04)	13.3 (11.8–15.15)	−1.486	0.137
Skeletal Muscle Mass	6.25 (5.017–7.388)	5.9 (5–6.95)	−1.48	0.139
Body Mass Index	17.03 ± 1.94	16.02 ± 1.54	4.338	**<0.001**
Percent Body Fat	22.75 (19.6–27.78)	23.5 (19.4–26.8)	−0.003	0.998

Bold values indicate *p* < 0.05.

## Data Availability

The datasets generated during and/or analyzed during the current study are available from the corresponding author on reasonable request. The data are not publicly available due to privacy concerns.

## Appendix A. Sleep Habits Questionnaire and Dietary Behavior Questionnaire

**Classification**	**Main Problems**	**Items**	**Option**
Sleep disturbances	Night awakening	Whether your child wakes up at night?	1. YES; 2. NO
	Daytime sleepiness	Whether your child has one of the following conditions? ① Inability to wake up spontaneously in the morning requires someone else to wake up. ② Waking up in the morning in a bad state. ③ Fatigue or sleepiness during the day while watching TV. ④ Fatigue or sleepiness during the day when travelling in a car.	1. YES; 2. NO
	Parasomnias	Whether your child has one of the following conditions? ① Grind teeth (during sleep). ② Talk in sleep. ③ Sleepwalking. ④ Have nightmares. ⑤ Restlessness (often with large body movements). ⑥ Snoring loudly. ⑦ Sleep apnea.	1. YES; 2. NO
	Sleep anxiety	Whether your child has one of the following conditions? ① Afraid of the dark. ② Crying before bedtime. ③ Afraid to sleep alone. ④ Difficulty sleeping in unfamiliar surroundings. ⑤ Moving into someone else’s bed in the middle of the night. ⑥ Waking up in the middle of the night crying and being difficult to calm. ⑦ Dyspnoea	1. YES; 2. NO
	Prolonged time to fall asleep	Is your child unable to fall asleep within 20 minutes of going to bed?	1. YES; 2. NO
	Poor sleep habits	Whether your child has one of the following conditions? ① Unable to sleep alone. ② Needs to be put to sleep.	1. YES; 2. NO
Dietary behavior	Diet partiality	Does your child have a significant diet partiality?	1. YES; 2. NO
		Please list the types of food your child resists eating.	Unrestricted options
